# Molecular Characterization of a Novel Family VIII Esterase with β-Lactamase Activity (*Ps*EstA) from *Paenibacillus* sp.

**DOI:** 10.3390/biom9120786

**Published:** 2019-11-26

**Authors:** Sena Kwon, Wanki Yoo, Young-Ok Kim, Kyeong Kyu Kim, T. Doohun Kim

**Affiliations:** 1 Department of Chemistry, College of Natural Science, Sookmyung Women’s University, Seoul 04310, Korea; mymy0445@naver.com (S.K.); vlqkshqk61@outlook.kr (W.Y.); 2 Department of Molecular Cell Biology, SungKyunKwan University School of Medicine, Suwon 2066, Korea; kyeongkyu@skku.edu; 3 Biotechnology Research Division, National Fisheries Research and Development Institute, Gijang, Busan 46083, Korea; yobest12@korea.kr

**Keywords:** *Ps*EstA, family VIII esterase, β-lactamase, antibiotics, immobilization

## Abstract

Molecular information about family VIII esterases, which have similarities with class C β-lactamases and penicillin-binding proteins, remains largely unknown. In this study, a novel family VIII esterase with β-lactamase activity (*Ps*EstA) from *Paenibacillus* sp. was characterized using several biochemical and biophysical methods. *Ps*EstA was effective on a broad range of substrates including tertiary butyl acetate, glyceryl tributyrate, glucose pentaacetate, olive oil, and *p*-nitrophenyl esters. Additionally, *Ps*EstA hydrolyzed nitrocefin, cefotaxime, and 7-aminocephalosporanic acid. Interestingly, two forms of immobilized *Ps*EstA (CLEAs-*Ps*EstA and mCLEAs-*Ps*EstA) showed high recycling property and enhanced stability, but hybrid nanoflowers (hNFs) of *Ps*EstA require improvement. This study provides a molecular understanding of substrate specificities, catalytic regulation, and immobilization of *Ps*EstA, which can be efficiently used in biotechnological applications.

## 1. Introduction

Bacterial β-lactamases hydrolyze chemical compounds containing a β-lactam ring and are the primary cause of bacterial resistance against different classes of β-lactam antibiotics. Therefore, the production of one or more types of β-lactamase is the most effective strategy through which clinically important gram-negative bacteria effectively hydrolyze the β-lactam rings of antibiotics, such as penicillins, cephalosporins, or carbapenems [1,2,3]. Based on amino acid motifs, protein sequence, molecular size, and 3D structure, β-lactamases are classified into four molecular classes termed A, B, C, and D [4]. Interestingly, classes A, C, and D hydrolyze their substrates through the formation of an acyl-enzyme with a catalytic serine, whereas class B utilizes a zinc ion to facilitate a hydrolytic reaction. Among β-lactamases, class C enzymes are of high importance, because they pose significant threats for antibiotic treatments due to their occurrence in many gram-negative pathogens, such as *Enterobacteriaceae* and *Pseudomonas* spp. [5,6]. Class C β-lactamases play a role in the resistance of these pathogens to cephalosporins, cephamycins, and carbapenems, and are not inhibited by clavulanic acid [7,8]. The molecular structure of class C β-lactamases consists of two domains of different sizes. The bigger domain contains a central antiparallel beta sheet flanked by alpha helices at each side, while the smaller domain is entirely composed of alpha helices and contains the catalytic serine residue [7,9,10,11].

There is an increasing need to comprehend class C β-lactamases at the molecular level to develop potent and novel inhibitors against them given that they are one of the major causes of antibiotic resistance in clinically significant bacterial pathogens. Interestingly, several esterases, collectively known as family VIII esterases, have recently been reported to have similarities with class C β-lactamases and hydrolyze nitrocefin or other β-lactam antibiotics [12,13,14,15]. These proteins have a Ser-Lys-Tyr catalytic triad, and their nucleophilic serine residue is present in the S-X-X-K motif located in the N-terminal region, instead of the G-X-S-X-G motif in most bacterial lipases/esterases [16,17]. A novel family VIII esterase with β-lactamase activity (*Ps*EstA) has recently been identified in *Paenibacillus* sp. PBS-2 [18]. Here, we biochemically characterized *Ps*EstA to lay the foundation for the understanding of this enzyme at the molecular level.

## 2. Materials and Methods

### 2.1. Sequence Analysis and Model Building

The primary sequences of *Ps*EstA and other related bacterial esterases/lipases, as well as class C β-lactamases, were retrieved from the NCBI database or Swiss-Prot. The phylogenetic tree was constructed using the neighbor-joining (NJ) method of MEGA 7.0 package [19]. Multiple sequence alignments were performed using Clustal Omega [20], and the results were rendered using ESPript [21]. The *Ps*EstA structure was modeled using the crystal structure of EstU1 from a metagenome (PDB code: 4IVK, 31% sequence identity) as a template on the SWISS-MODEL server. Molecular docking was performed using the flexible side chain method [22,23]. The coordinate of the tetrahedral intermediate was built using the PRODRG server [24]. Processing of *Ps*EstA and the tetrahedral intermediate was performed with AutoDock Vina [25]. Graphical representations were prepared using PyMOL software [26].

### 2.2. Protein Purification

Cloning and expression of *Ps*EstA were performed as previously described [18]. Transformed *E. coli* BL21(λDE3) cells were grown in LB medium until OD_600_ reached 0.4–0.6. After incubation with 1 mM isopropyl-β-d-1-thiogalactoside for 4 h, the cells were harvested by centrifugation at 6000 rpm and at 4 °C for 20 min, and then resuspended in lysis buffer (20 mM Tris-HCl, 20 mM imidazole, and 1 mM EDTA; pH 7.4). Next, the cell suspension was lysed by sonication and cell debris were precipitated out by centrifugation at 20,000 rpm for 30 min. The supernatants were then loaded onto HisTrap HP columns in an AKTA Prime Plus Liquid Chromatography System (GE Healthcare, Chicago, IL, USA). After extensive washing, the recombinant *Ps*EstA protein was eluted using an imidazole gradient, and the pooled fractions were buffer-exchanged into a storage buffer (20 mM Tris-HCl and 1 mM EDTA; pH 8.0) without cleaving the N-terminal His-tag. Protein purity and molecular weight were confirmed by SDS-PAGE. Protein concentrations were determined using a Bio-Rad Protein assay kit (Bio-Rad Laboratories, Hercules, CA, USA).

### 2.3. Characterization of PsEstA

Activity staining was performed using native-PAGE followed by staining of the gel with Coomassie Brilliant Blue R-250 and 4-methylumbelliferone (4-MU) acetate [27,28]. For the overlay activity assay, native-PAGE analysis was performed. Subsequently, the gel was washed three times with a storage buffer, and then incubated with 4-methylumbelliferyl (4-MU) acetate to detect the fluorescent signal under UV illumination [16,17]. Hydrolysis of 4-methylumbelliferone (4-MU) acetate or phosphate by *Ps*EstA was also investigated in an Eppendorf tube in an ultraviolet (UV) illumination box. For gel filtration analysis, purified *Ps*EstA was applied onto Superdex 200GL columns in AKTA UPC-900 system (GE Healthcare, Chicago, IL, USA). Matrix-assisted laser desorption time-of-flight mass spectrometry was performed in the positive ion mode on a Voyager™ BioSpectrometry™. Urea-induced unfolding was performed following 1 h incubation of *Ps*EstA with urea at 25 °C. The emission spectra from 300 nm to 400 nm were recorded after excitation at 295 nm. Thermal unfolding was monitored from 15 °C to 90 °C at 222 nm using circular dichroism (CD) signals with a thermostatic cell holder.

The esterase activity of *Ps*EstA and its S58A mutant was analyzed using *p*-nitrophenyl (*p*-NP) esters and naphthyl esters. Specifically, substrates included *p*-nitrophenyl acetate (*p-*NA), *p*-nitrophenyl butyrate (*p-*NB), *p*-nitrophenyl hexanoate (*p-*NH), *p*-nitrophenyl octanoate (*p-*NO), *p*-nitrophenyl decanoate (*p-*ND), and *p*-nitrophenyl phosphate (*p-*NPP). For naphthyl esters, 1-naphthyl acetate (1-NA), 2-naphthyl acetate (2-NA), and 1-naphthyl butyrate (1-NB) were used. The effects of the chemicals (urea, NaCl, glycerol, Triton X-100, and Tween 20) on the activity of *Ps*EstA were investigated after 1 h incubation of each chemical with *Ps*EstA. The standard assay solution included 5 μM of *p-*NB in 20 mM Tris-HCl (pH 8.0) with 0.5 μg *Ps*EstA, and the assay was run for 5 min at 25 °C. All the assays described above were performed using an Epoch 2 Microplate spectrophotometer (BioTek, Winooski, VT, USA) and the enzyme activity of *Ps*EstA in buffer alone was defined as 100%.

### 2.4. pH Indicator-Based Hydrolysis Assay

For pH indicator-based colorimetric assays, *Ps*EstA was added into a phenol red-containing substrate solution [27,28,29]. For these assays, 10 μg of *Ps*EstA was added to substrate solutions at 37 °C containing 0.13 g·L^−1^ phenol red. The substrates included glyceryl lipids (glyceryl tributyrate and glyceryl trioleate), natural oils (olive oil and fish oil), tertiary alcohol esters (tertiary butyl acetate, α-terpinyl acetate, and linalyl acetate), and carbohydrate esters (glucose pentaacetate, cellulose acetate, and glucosamine acetate). A rhodamine B–olive oil (or fish oil) mixture was prepared, and the fluorescence spectra were recorded from 500 nm to 600 nm after excitation at 350 nm [30]. For enantioselectivity analysis, *Ps*EstA was incubated with enantiomeric solutions containing 300 mM methyl-(*R*)-(−)-3-hydroxy-2-methylpropionate or methyl-(*S*)-(+)-3-hydroxy-2-methylpropionate.

### 2.5. β-Lactamase Assay

The β-lactamase activity of *Ps*EstA was determined using the chromogenic β-lactam substrate nitrocefin [17]. *Ps*EstA (0.1 mg/mL) was reacted with a 484 µM nitrocefin solution in 20 mM Tris-HCl pH 8.5 at 25 °C, and the color change in the reaction mixture was observed. In addition, pH indicator-based colorimetric assay was performed to investigate the activity of *Ps*EstA on 1 mM cefotaxime (CTX) and 7-aminocephalosporanic acid (7-ACA) at 25 °C for 1 h.

### 2.6. Immobilization of PsEstA

For preparing crosslinked enzyme aggregates (CLEAs), 0.5 mg·mL^−1^ of *Ps*EstA was coprecipitated by 80% (*w*/*v*) ammonium sulfate at 4 °C. Crosslinking was then performed by the dropwise addition of glutaraldehyde to the final concentration of 25 mM at room temperature. After an overnight incubation followed by centrifugation at 15,000 *g* for 10 min, the pellet (CLEA-*Ps*EstA) was resuspended and repeatedly washed until no activity was observed in the supernatant. The preparation and addition of the magnetic Fe_3_O_4_ nanoparticles were carried out as previously described [27,31]. Nanoparticles were mixed with *Ps*EstA solution containing ammonium sulfate and glutaraldehyde. After gentle agitation for 12 h, magnetic CLEAs of *Ps*EstA (mCLEA-*Ps*EstA) were precipitated by centrifugation at 15,000× *g* for 10 min and stored in 50 mM sodium phosphate buffer (pH 7.5) at 4 °C. Scanning electron microscope images were obtained at various magnifications (50,000–100,000×) using a Carl Zeiss SUPRA 55VP microscope. The chemical stabilities of the three forms of *Ps*EstA (free *Ps*EstA, CLEA-*Ps*EstA, and mCLEA-*Ps*EstA) were determined by measuring the residual activity toward *p*-nitrophenyl butyrate (*p*-NB) after 1 h incubation in the presence of the chemical compounds [ethanol, *iso*-propanol, sodium dodecyl sulfate (SDS), urea] at 25 °C. For the recycling process, the two immobilized forms of *Ps*EstA (CLEA-*Ps*EstA and mCLEA-*Ps*EstA) were extensively washed and then reused in the next cycle. For preparing enzyme-inorganic hybrid nanoflower (hNF), *Ps*EstA (0.05, 0.1, and 0.5 mg·mL^−1^) was added into 800 μM of the Cu^2+^ metal solutions for a total volume of 3 mL [32]. The resulting mixtures were incubated at 25 °C and centrifuged at 15,000× *g* for 10 min. The pellets were resuspended and transferred to 1.5 mL microtubes. In the reusability tests, hNF-*Ps*EstA was recovered by centrifugation and extensively washed before being reused in the next cycle. Then, fresh substrate of 1 mM *p*-nitrophenyl butyrate (*p*-NB) was reacted for another cycle and activity was measured. In these experiments, the enzyme activity of free *Ps*EstA was defined as 100%.

## 3. Results and Discussion

### 3.1. Bioinformatic Analysis

**Figure 1:**
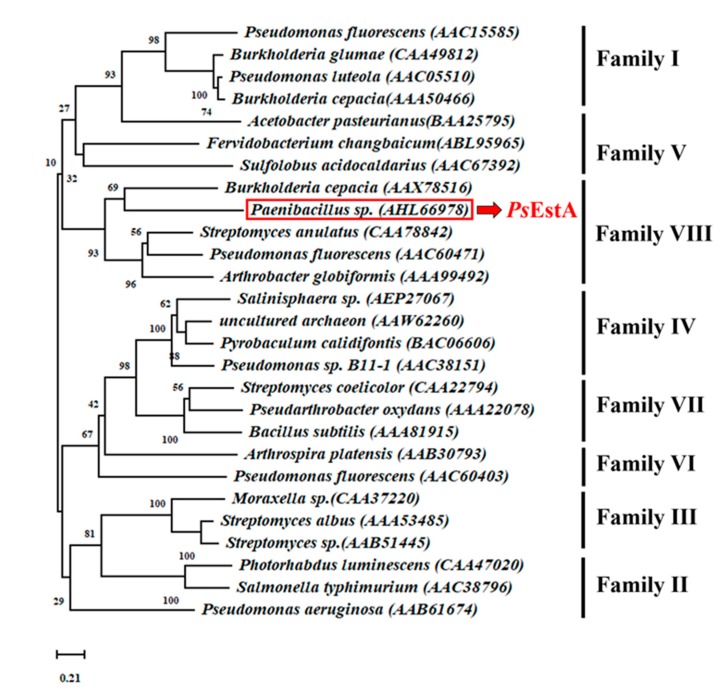
A phylogenetic tree analysis of *Ps*EstA. The phylogenetic tree was constructed with MEGA v 7.0 using neighbor-joining method with 3000 iterations.

For phylogenetic tree analysis of *Ps*EstA, the 26 representative bacterial lipases/esterases sequences were analyzed. As shown in Figure 1, *Ps*EstA belongs to family VIII esterases, which include EstC [12], Est-Y29 [33], EstB [34], and EstU1 [35].

**Figure 2:**
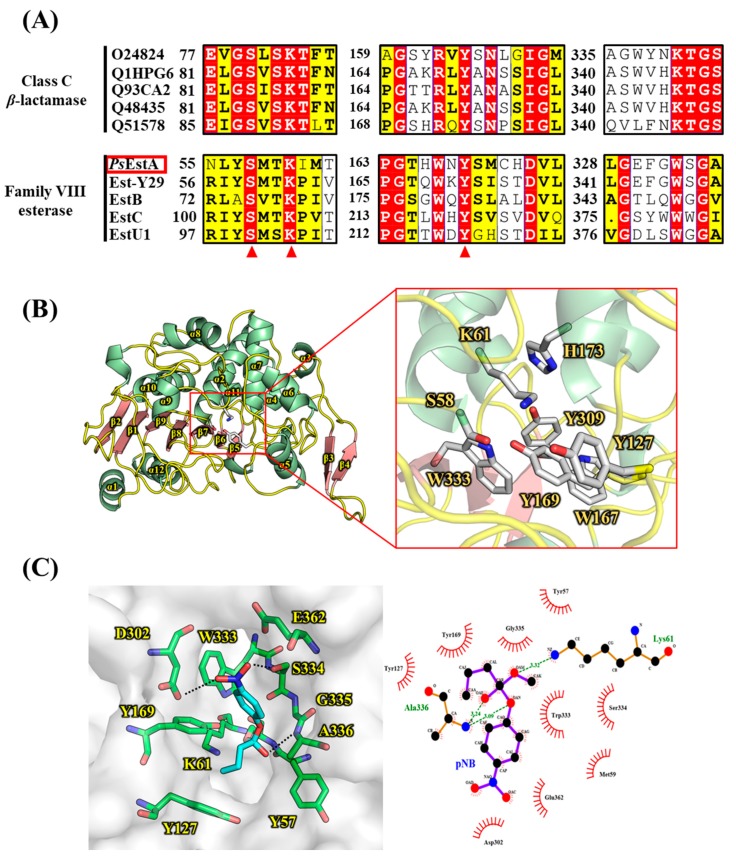
Multiple sequence alignment and structural representation of *Ps*EstA. (**A**) Multiple sequence alignment was performed among class C β-lactamases and family VIII esterases. Three characteristic motifs (motif I, II, and III) with important catalytic residues are shown. Highly conserved catalytic residues are shown in red triangles. (**B**) A molecular model of *Ps*EstA is shown in ribbon diagram, and α-helices and β-strands of *Ps*EstA are represented in light green and light red, respectively. Catalytic residues and putative substrate binding pocket residues are shown as a ball-and-stick model. (**C**) (left) Modeling of the complex formation of *p*-nitrophenyl butyrate (*p*NB, cyan) in the pocket of *Ps*EstA. The amino acid residues interacting with *p*NB are shown as sticks (green). (right) LigPlot analysis between *p*-nitrophenyl butyrate and *Ps*EstA.

As shown in Figure 2A, three motifs were found to be highly conserved among class C β-lactamases based on multiple sequence alignment. These three motifs (motif I, II, and III) have also been identified in family VIII esterases. In motif I, a nucleophilic Ser^58^ is located in the characteristic S^58^-x-x-K^61^ tetrapeptide, and the alkoxide ion formation at Ser^58^ is believed to be enhanced by Lys^61^. The Tyr^172^ residue in motif II is involved in the substrate recognition and specificity regulation. Although motif III of class C β-lactamases is composed of the highly conserved K-T-G sequence, a W-x-G motif is observed instead in *Ps*EstA and other family VIII esterases. The third residue of motif III has to be glycine because otherwise, other amino acids would be sterically unfavorable for the binding of the substrate. In *Ps*EstA, the W-S-G sequence was observed in motif III. In sequence analysis, *Ps*EstA was found to contain a higher percentage of acidic (Asp and Glu, 12.0%) than basic (Lys and Arg, 9.3%) amino acids.

**Figure 3:**
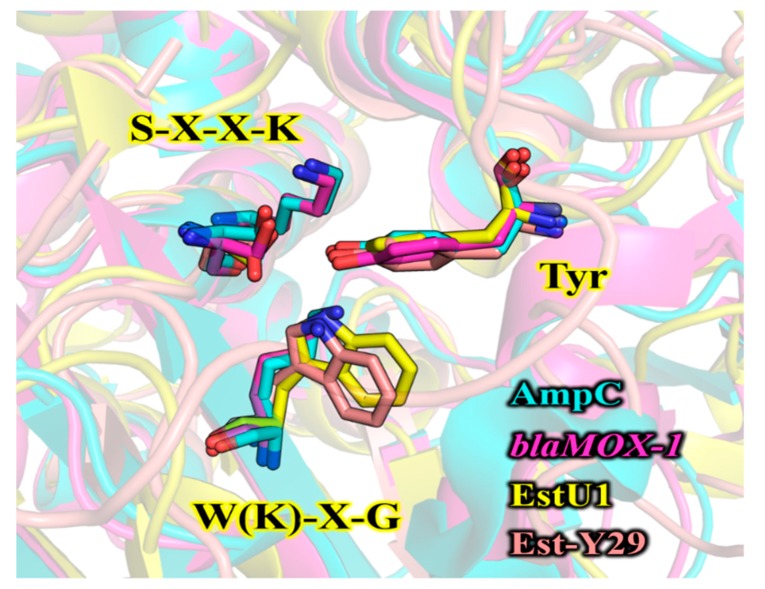
Structural comparison of the catalytic amino acids between class C *β*-lactamases and family VIII esterases. The key residues in three motifs are shown in sticks. Two class C *β*-lactamases [AmpC (PDB code: 1S6R) and blaMOX-1 (PDB code: 3W8K)] and two family VIII esterases [EstU1 (PDB code: 4IVI) and Est-Y29 (PDB code: 4P6B)] are colored in cyan, magenta, yellow, and pink, respectively. The structure superimposition was performed using PyMOL software.

The structural model of *Ps*EstA consisted of a large α/β domain and a small α-helical domain, and these features are frequently observed in other β-lactamases and family VIII esterases [14,17,34,35]. The nucleophilic serine was found to be located near the surface of the tunnel formed by two domains (Figure 2B). The putative catalytic triad made of Ser^58^, Lys^61^, and Tyr^169^ is positioned within a catalytic pocket close to the surface (Figure 2B, rectangular region). The substrate-binding pocket is mainly delineated by the aromatic amino acids Tyr^127^, Tyr^169^, and Trp^333^, presumably regulating the entrance of substrates via hydrophobic interactions. These residues are also highly conserved in most family VIII esterases. In molecular docking analysis, Tyr^169^ and Trp^333^ were shown to stabilize the *p*-nitrophenol ring, while side chains of Asp^302^ and Ser^334^ formed hydrogen bonds with the nitro moiety (-NO_2_) of *p*-nitrophenyl butyrate (Figure 2C). In addition, backbone nitrogen of Ala^336^ is involved in the formation of an oxyanion hole. This model analysis suggested a number of active-site residues seem to be involved in hydrogen bonding networks among other conserved residues or substrate [34]. In addition to conservation of primary sequences, spatial orientation of catalytic residues between class C *β*-lactamases and family VIII esterases are very similar. As shown in Figure 3, catalytic residues of *Ps*EstA have comparable conformation to those of AmpC [36] and blaMOX-1 [37], as well as EstU1 and Est-Y29.

### 3.2. Characterization of PsEstA

**Figure 4:**
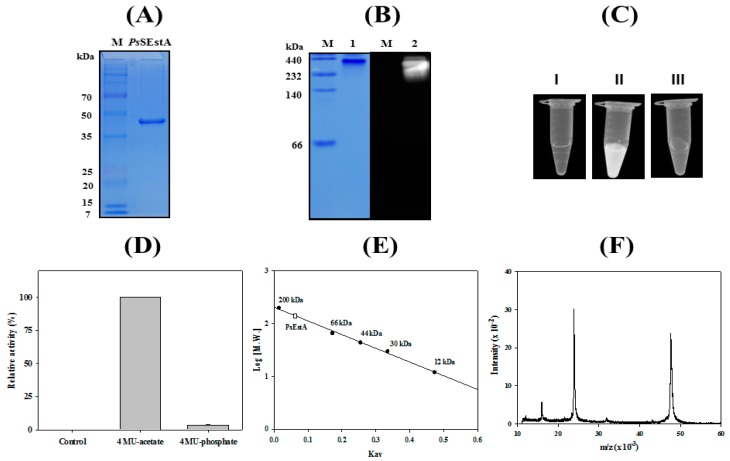
Molecular characterization of *Ps*EstA. (**A**) Purified *Ps*EstA was analyzed by SDS-PAGE. (**B**) 4-MU overlay assay of *Ps*EstA. CBB staining (left) and fluorescence (right). (**C**) Hydrolysis of 4-methylumbelliferyl (4-MU) acetate and -phosphate. *Ps*EstA only (I), 4-MU acetate with *Ps*EstA (II), and 4-MU phosphate with *Ps*EstA (III). (**D**) Fluorescence intensity of *Ps*EstA only, 4-MU acetate with *Ps*EstA, and 4-MU phosphate with *Ps*EstA were measured. (**E**) Gel filtration analysis of *Ps*EstA. The column was calibrated with β-amylase (200 kDa), BSA (66 kDa), peroxidase (44 kDa), carbonic anhydrase (30 kDa), and cytochrome C (12 kDa). (**F**) Mass analysis of *Ps*EstA. The [M + H]^+^ ion peak was observed at *m*/*z* value of 47,738.

The recombinant *Ps*EstA was highly purified using an immobilized metal-affinity column, and a single band was observed (Figure 4A). The molecular mass of *Ps*EstA was estimated to be approximately 45 kDa by SDS-PAGE, and this value is similar to those of Est-Y29 [33] and EstB [34], but smaller than EstU1 [35]. An overlay activity assay showed high fluorescence at the position where purified *Ps*EstA was located in native PAGE (Figure 4B). Furthermore, strong fluorescence intensity was observed for 4-MU acetate and *Ps*EstA, but not for 4-MU phosphate and *Ps*EstA (Figure 4C,D). In native PAGE, the molecular mass of active *Ps*EstA was found to be substantially greater than 200 kDa, implying that *Ps*EstA is in an oligomeric conformation. A similar behavior indicating an oligomeric state of *Ps*EstA was also observed in gel filtration chromatography (Figure 4E), which has been reported for other family VIII esterases [38,39,40]. The mass spectrometric analysis showed a major peak (*m*/*z*) at 47.7 kDa with all additional amino acids including the His-tag (Figure 4F).

### 3.3. Biochemical Assay

**Figure 5:**
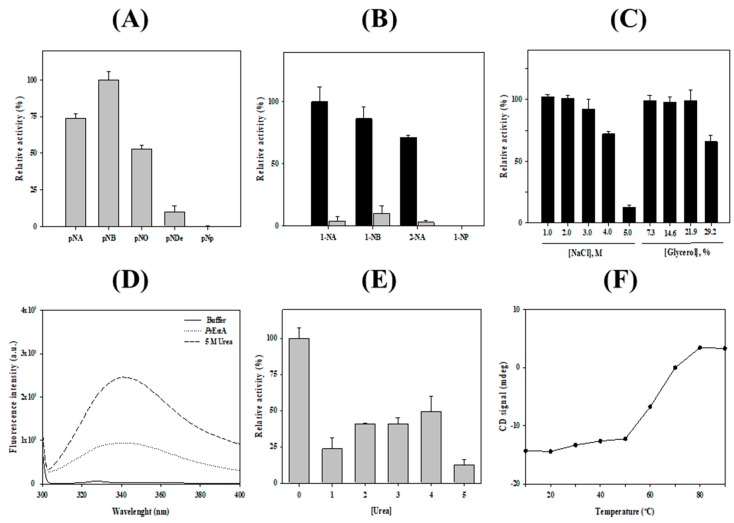
Biochemical properties of *Ps*EstA. (**A**) Substrate specificity of *Ps*EstA toward using *p*-nitrophenyl esters (acetate: *p-*NA; butyrate: *p-*NB; octanoate: *p-*NO; decanoate: *p-*NDe; phosphate: *p-*Np). The hydrolase activities are shown relative to the activity toward *p-*NB. (**B**) Regioselectivity of *Ps*EstA toward naphthyl esters. The hydrolase activities are shown relative to the activity toward 1-NA. Activities of wild-type *Ps*EstA (black) and S58A (gray) are shown for comparison. (**C**) Activity of *Ps*EstA toward *p-*NB in the presence of NaCl (left) and glycerol (right). (**D**) Intrinsic fluorescence spectra of *Ps*EstA with or without 5 M urea. (**E**) Effects of urea on the activity of *Ps*EstA. (**F**) Thermal unfolding of *Ps*EstA was monitored by circular dichroism at 222 nm from 10 to 90 °C. The standard assay solution included 1 mM *p*-nitrophenyl butyrate (*p*-NB) as a substrate in 20 mM Tris-HCl (pH 8.0).

The hydrolytic activity of *Ps*EstA was analyzed using *p*-nitrophenyl (*p*-NP) esters with acyl chain lengths of different length [27,28,29,41]. As shown in Figure 5A, *Ps*EstA had a strong substrate preference for *p*-NA or *p*-NB. However, almost no enzyme activity was observed for the long-chain substrates *p*-NO and *p*-ND. Similarly, other members of family VIII esterases such as Est22 [13], EstU1 [42], or EstM-N1 [43] showed substrate preference for *p*-NB. When naphthyl esters were used as the substrates, the highest enzymatic activities were observed with 1-naphthyl acetate (1-NA), followed by 1-naphthyl butyrate (1-NB) and 2-naphthyl acetate (2-NA). However, mutation of Ser^58^ residue abolished most of the hydrolytic activity (Figure 5B). As shown in Figure 5B, *Ps*EstA exhibited approximately 75% activity on 2-NA. In contrast, it showed almost no activity on 1-naphthyl phosphate (1-NP). *Ps*EstA was also shown to be highly stable in the presence of NaCl and glycerol. Specifically, it was shown to retain approximately 100% and 70% of its enzymatic activity in the presence of 3.0 M and 4.0 M NaCl, respectively (Figure 5C). In addition, *Ps*EstA exhibited strong tolerance to glycerol with almost no loss of enzymatic activity after incubating with up to 30% glycerol.

The chemical stability of *Ps*EstA was investigated by monitoring the intrinsic fluorescence spectra. In its native form, *Ps*EstA exhibited a λ_max_ at 334 nm, implying that all the tryptophan residues of *Ps*EstA were located in the hydrophobic interior. However, a red shift of λ_max_ to 344 nm was observed with a remarkable increase in fluorescence intensity in the presence of 5 M urea, suggesting that the tryptophan residues were mostly exposed to the solvent (Figure 5D). The chemical stability of *Ps*EstA in urea was also investigated by measuring the enzymatic activity in the presence of increasing concentrations of urea. At 4.0 M, ~50% of the initial activity was retained (Figure 5E). Next, the thermal stability of *Ps*EstA was investigated by monitoring its thermal denaturation from 15 to 90 °C using far-UV CD at 222 nm. *Ps*EstA showed only minor changes up to 50 °C, and its melting temperature was determined as 63 °C (Figure 5F).

### 3.4. Substrate Analysis

**Figure 6:**
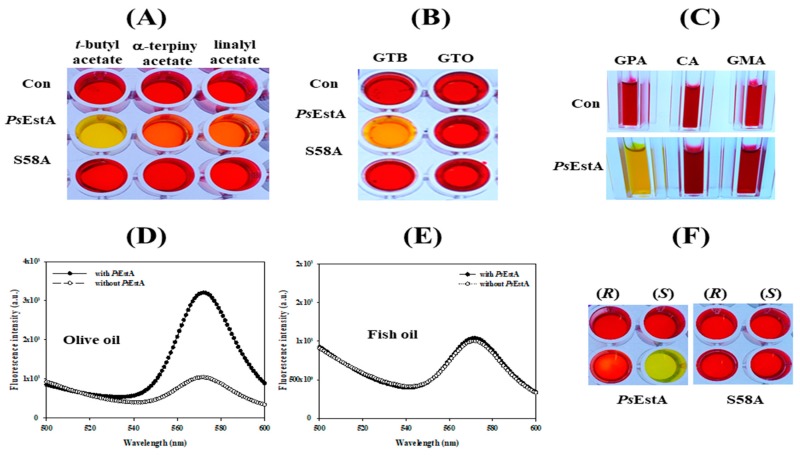
Substrate analysis of *Ps*EstA. The pH shift assay was carried out toward (**A**) acetylated carbohydrates (GPA: glucose pentaacetate; CA: cellulose acetate; NAG: *N*-acetyl-glucosamine), (**B**) triacyl glycerols (GTB:glyceryl tributyrate; GTO:glyceryl trioleate), (**C**) tertiary alcohol esters (*tert*-butyl acetate, α-terpinyl acetate, and linalyl acetate). Hydrolysis of olive oil (**D**) and fish oil (**E**) was investigated using fluorescence spectra. The excitation wavelength was set at 350 nm, and emission was recorded from 500 to 600 nm. (**F**) Enantioselectivity of *Ps*EstA with methyl-(*R*)-(−)-3-hydroxy-2-methylpropionate or methyl-(*S*)-(+)-3-hydroxy-2-methylpropionate.

The hydrolytic properties of *Ps*EstA for acetylated carbohydrates, tertiary alcohol esters, and lipids were evaluated using a colorimetric assay [28,29,41]. The tertiary alcohol esters *tert*-butyl acetate, α-terpinyl acetate, and linalyl acetate were used. As shown in Figure 6A, *Ps*EstA could effectively hydrolyze *tert*-butyl acetate, but not linalyl acetate or α-terpinyl acetate. Additionally, a significant hydrolytic activity of *Ps*EstA was detected only for glyceryl tributyrate based on the yellow color of the solution (Figure 6B). Furthermore, *Ps*EstA displayed a high activity for glucose pentaacetate, although no significant activity was detected for cellulose acetate or glucosamine acetate (Figure 6C). In addition, a high level of enzymatic activity of *Ps*EstA was observed toward olive oil, judging from the fluorescence spectra (Figure 6D,E). For enantioselectivity analysis, (*R*)- and (*S*)-methyl-β-hydroxyisobutyrate were used [27,32]. Following incubation with *Ps*EstA, only the reaction mixture containing the (*S*)-enantiomer turned yellow, showing the (*S*)-selectivity of *Ps*EstA (Figure 6F). This indiscriminate nature of *Ps*EstA could be useful for industrial applications as a biocatalyst.

### 3.5. β-Lactamase Activity of PsEstA

**Figure 7:**
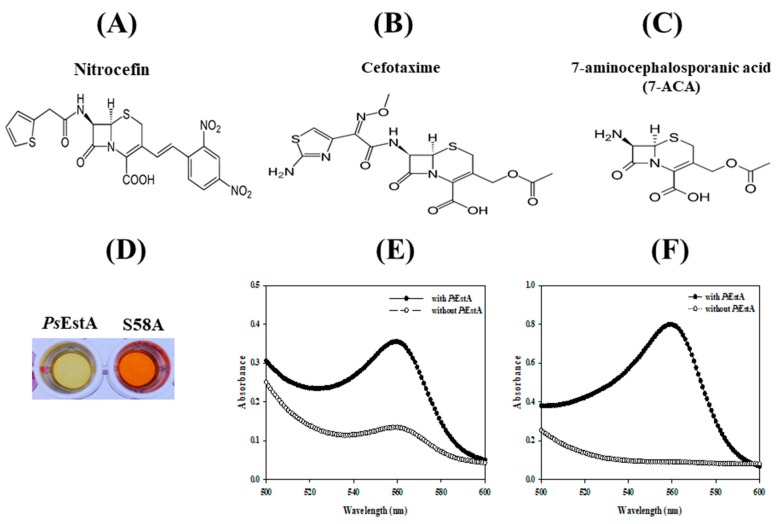
Activity of *Ps*EstA toward β-lactam compounds. Chemical structure of nitrocefin (**A**), cefotaxime (**B**), and 7-aminocephalosporanic acid (**C**) are shown. The β-lactamase activity of *Ps*EstA and its S58A mutant was investigated using pH shift assay. (**D**) Color change of nitrocefin-containing solution with *Ps*EstA or S58A mutant. Absorbance spectra were recorded in cefotaxime (**E**) and 7-aminocephalosporanic acid (7-ACA) (**F**) with or without *Ps*EstA.

To investigate the β-lactamase activity of *Ps*EstA, a chromogenic β-lactamase substrate (nitrocefin) was used as a substrate [12,17]. As shown in Figure 7, *Ps*EstA apparently showed β-lactamase activity toward nitrocefin. The β-lactamase activity of *Ps*EstA was further investigated using 7-ACA and CTX. *Ps*EstA showed significant catalytic activities toward cefotaxime (CTX) and 7-aminocephalosporanic acid (7-ACA) in a pH shift assay (Figure 7E,F). In accordance with *Ps*EstA, Est22 and EstU1 were shown to possess a noteworthy β-lactam hydrolytic activity [13,35]. In contrast, EstC showed a significant hydrolyzing activity for nitrocefin, but none of these esterases displayed any activity for other β-lactam substrates [12].

### 3.6. Immobilization of PsEstA

**Figure 8:**
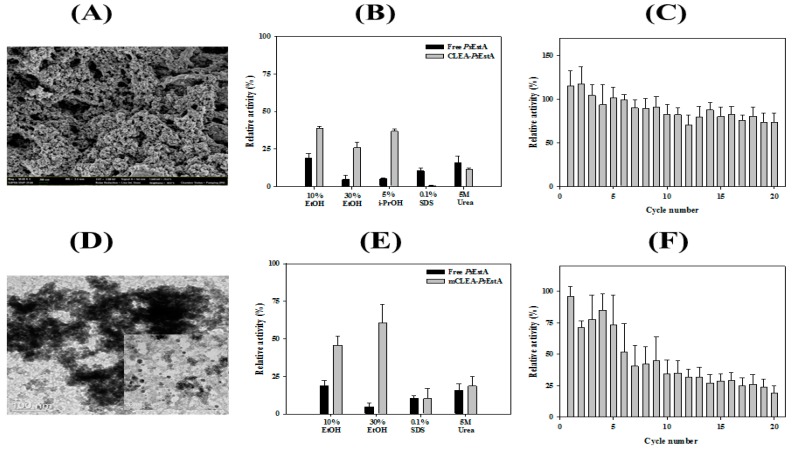
Characterization of immobilized *Ps*EstA. (**A**) Field emission scanning electron microscopic (FE-SEM) images of crosslinked enzyme aggregates (CLEAs). (**B**) Chemical stabilities of free *Ps*EstA and CLEAs-*Ps*EstA were compared to ethanol, *iso*-propanol, SDS, and 5 M urea. (**C**) Reusability of CLEAs-*Ps*EstA was investigated for 20 cycles. (**D**) Transmission electron microscopic images of magnetic nanoparticles. (**E**) Chemical stabilities of free *Ps*EstA and mCLEA-*Ps*EstA. (**F**) Reusability of mCLEA-*Ps*EstA was investigated for 20 cycles. In these experiments, the enzyme activity of *Ps*EstA in buffer alone was defined as 100%. The standard assay solution included 1 mM *p*-nitrophenyl butyrate (*p*-NB) as a substrate in 20 mM Tris-HCl (pH 8.0).

Enzyme immobilization has been explored in a large variety of industrial applications [44,45]. Crosslinked enzyme aggregates (CLEAs) constitute one of the highly studied methods to generate highly efficient immobilized biocatalysts [46]. Toward this end, CLEAs-*Ps*EstA was prepared by precipitating *Ps*EstA with ammonium sulfate and glutaraldehyde. The SEM images of CLEAs-*Ps*EstA showed formation of globular structures with a diameter of 1–2 nm (Figure 8A). The stabilities of both free *Ps*EstA and CLEAs-*Ps*EstA in the presence of several chemical compounds were compared (Figure 8B). For 5 M urea and 0.1% (*v*/*v*) SDS, there were no significant differences between these two forms. However, for alcohols such as ethanol and *iso*-propanol, CLEAs-*Ps*EstA was highly active compared with the free form of the enzyme. Specifically, with 30% ethanol, free *Ps*EstA retained only ~3% of its original activity, whereas CLEAs-*Ps*EstA showed ~35% of the original activity. The operational stability of CLEAs-*Ps*EstA was studied up to 20 cycles. As shown in Figure 8C, CLEAs-*Ps*EstA was highly stable for 20 cycles, retaining at the end ~75% of the original activity.

Furthermore, *Ps*EstA was immobilized as CLEAs on magnetite nanoparticles for an efficient separation during industrial applications [27,32]. To obtain magnetic CLEA form of *Ps*EstA (mCLEA- *Ps*EstA), *Ps*EstA was coaggregated with nanoparticles, and then chemically crosslinked using glutaraldehyde. Transmission electron microscopy showed that the magnetic nanoparticles had a diameter of 5–8 nm (Figure 8D). The effects of the chemical compounds on the activities of mCLEA-*Ps*EstA and free *Ps*EstA were investigated by measuring the residual activities in the presence of each chemical. As shown in Figure 8E, the enzymatic activities of mCLEA-*Ps*EstA were largely remarkably higher than those of the free form of *Ps*EstA in the presence of the alcohols tested. Specifically, free *Ps*EstA showed a relative activity of 21% in the presence of 10% EtOH, whereas mCLEA-*Ps*EstA retained 47% of the initial activity. Moreover, although the free *Ps*EstA retained less than 5% of its original activity in the presence of 30% EtOH, mCLEA-*Ps*EstA showed 62% activity at the end. However, even 0.1% (*v*/*v*) SDS was sufficient to inactivate both free *Ps*EstA and mCLEA-*Ps*EstA almost completely. Similar behavior was also observed in the presence of 5 M urea. In reusability analysis, mCLEA-*Ps*EstA retained ~75% of its original activity after the 4th cycle, but lost a substantial level of activity at the 5th cycle (Figure 8F). Taken together, immobilization of *Ps*EstA (CLEAs-*Ps*EstA and mCLEAs-*Ps*EstA) was effectively performed, and these immobilized forms could function more effectively than the free form of *Ps*EstA.

### 3.7. Formation of Organic–Inorganic Hybrid PsEstA Nanoflower

**Figure 9:**
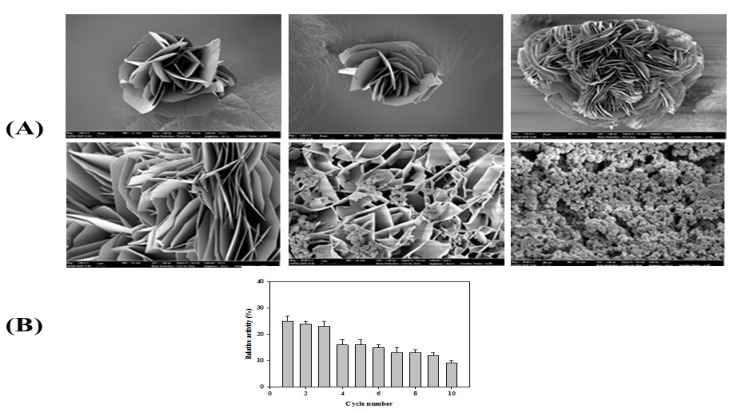
Properties of *Ps*EstA-hNFs. (**A**) Field emission scanning electron microscopic images of *Ps*EstA-hNFs at different magnifications. (**B**) Reusability of mCLEA-*Ps*EstA was investigated for 10 cycles. The standard assay solution included 1 mM *p*-nitrophenyl butyrate (*p*-NB) as a substrate in 20 mM Tris-HCl (pH 8.0).

Recently, syntheses of organic–inorganic hybrid nanoflowers (hNFs) with greatly enhanced catalytic activities and stabilities have been reported [47,48]. Herein, the formation and catalytic activity of hybrid nanoflowers containing *Ps*EstA were pursued. Scanning electron microscopy showed that the hybrid nanoflowers of *Ps*EstA were hierarchical peony-like structures assembled from interlaced nanoplates (Figure 9A). Reusability tests were also carried out for 10 cycles showing that the residual activity was ~25% by the 3rd cycle (Figure 9B).

## 4. Conclusions

Although family VIII esterases with β-lactamase activity have attracted considerable research interest, there is still limited information available regarding this enzyme family. Here, a novel family VIII esterase with β-lactamase activity (*Ps*EstA) from *Paenibacillus* sp. was characterized using molecular modeling, spectroscopic methods, biochemical assays such as β-lactamase assay, and immobilization strategies. This structural and functional characterization of *Ps*EstA is expected to provide a molecular platform for the comprehensive understanding of family VIII esterases as well as β-lactamase, although the physiological role of *Ps*EstA still needs to be explored. In addition, considering the fact that very few enzymes of this family have been biochemically characterized and immobilized for biotechnological applications, *Ps*EstA could be a promising target for biotechnological applications [49,50,51]. Further studies on *Ps*EstA including mutagenesis of the key residues, in-depth kinetic analysis, and evaluation of enzyme–substrate complex formation will be necessary for a thorough understanding of this enzyme at the molecular level.

## References

[1] Fisher J.F., Meroueh S.O., Mobashery S. (2005). Bacterial resistance to beta-lactam antibiotics: Compelling opportunism, compelling opportunity. Chem. Rev..

[2] Salahuddin P., Kumar A., Khan A.U. (2018). Structure, function of serine and metallo-β-lactamases and their inhibitors. Curr. Protein Pept. Sci..

[3] Bush K. (2013). Proliferation and significance of clinically relevant β-lactamases. Ann. N. Y. Acad. Sci..

[4] Bush K., Jacoby G.A. (2010). Updated functional classification of beta-lactamases. Antimicrob. Agents Chemother..

[5] Bush K. (2018). Past and present perspectives on β-lactamases. Antimicrob. Agents Chemother..

[6] Powers R.A. (2016). Structural and functional aspects of extended spectrum AmpC cephalosporinases. Curr. Drug Targets..

[7] Pozzi C., Di Pisa F., De Luca F., Benvenuti M., Docquier J.D., Mangani S. (2018). Atomic-resolution structure of a class C β-lactamase and its complex with Avibactam. ChemMedChem.

[8] Tooke C.L., Hinchliffe P., Bragginton E.C., Colenso C.K., Hirvonen V.H.A., Takebayashi Y., Spencer J. (2019). β-Lactamases and β-Lactamase Inhibitors in the 21st Century. J. Mol. Biol..

[9] Na J.H., Cha S.S. (2016). Structural basis for the extended substrate spectrum of AmpC BER and structure-guided discovery of the inhibition activity of citrate against the class C β-lactamases AmpC BER and CMY-10. Acta Crystallogr. D Struct. Biol..

[10] Oguri T., Ishii Y., Shimizu-Ibuka A. (2015). Conformational change observed in the active site of class C β-lactamase MOX-1 upon binding to aztreonam. Antimicrob. Agents Chemother..

[11] Bhattacharya M., Toth M., Antunes N.T., Smith C.A., Vakulenko S.B. (2014). Structure of the extended-spectrum class C β-lactamase ADC-1 from *Acinetobacter baumannii*. Acta Crystallogr. D Biol. Crystallogr..

[12] Rashamuse K., Magomani V., Ronneburg T., Brady D. (2009). A novel family VIII carboxylesterase derived from a leachate metagenome library exhibits promiscuous beta-lactamase activity on nitrocefin. Appl. Microbiol. Biotechnol..

[13] Mokoena N., Mathiba K., Tsekoa T., Steenkamp P., Rashamuse K. (2013). Functional characterisation of a metagenome derived family VIII esterase with a deacetylation activity on β-lactam antibiotics. Biochem. Biophys. Res. Commun..

[14] Cha S.S., An Y.J. (2016). Crystal structure of EstSRT1, a family VIII carboxylesterase displaying hydrolytic activity toward oxyimino cephalosporins. Biochem. Biophys. Res. Commun..

[15] Lee H.W., Jung W.K., Kim Y.H., Ryu B.H., Kim T.D., Kim J., Kim H. (2016). Characterization of a novel alkaline family VIII esterase with *S*-enantiomer preference from a compost metagenomic library. J. Microbiol. Biotechnol..

[16] Ngo T.D., Ryu B.H., Ju H., Jang E.J., Kim K.K., Kim T.D. (2014). Crystallographic analysis and biochemical applications of a novel penicillin-binding protein/β-lactamase homologue from a metagenomic library. Acta Crystallogr. D Biol. Crystallogr..

[17] Ryu B.H., Ngo T.D., Yoo W., Lee S., Kim B.Y., Lee E., Kim K.K., Kim T.D. (2016). Biochemical and structural analysis of a novel esterase from *Caulobacter crescentus* related to penicillin-binding protein (PBP). Sci. Rep..

[18] Kim Y.O., Park I.S., Nam B.H., Kim D.G., Jee Y.J., Lee S.J., An C.M. (2014). A novel esterase from *Paenibacillus* sp. PBS-2 is a new member of the β-lactamase belonging to the family VIII lipases/esterases. J. Microbiol. Biotechnol..

[19] Kumar S., Stecher G., Tamura K. (2016). MEGA 7: Molecular evolutionary genetics analysis version 7.0 for bigger datasets. Mol. Biol. Evol..

[20] Sievers F., Higgins D.G. (2018). Clustal Omega for making accurate alignments of many protein sequences. Protein Sci..

[21] Gouet P., Robert X., Courcelle E. (2003). ESPript/ENDscript: Extracting and rendering sequence and 3D information from atomic structures of proteins. Nucleic Acids Res..

[22] Bianco G., Forli S., Goodsell D.S., Olson A.J. (2016). Covalent docking using autodock: Two-point attractor and flexible side chain methods. Protein Sci..

[23] Morris G.M., Huey R., Lindstrom W., Sanner M.F., Belew R.K., Goodsell D.S., Olson A.J. (2009). AutoDock4 and AutoDockTools4: Automated docking with selective receptor flexibility. J. Comput. Chem..

[24] Schüttelkopf A.W., Van Aalten D.M. (2004). PRODRG: A tool for high-throughput crystallography of protein–ligand complexes. Acta Crystallogr. D Struct. Biol..

[25] Trott O., Olson A.J. (2010). AutoDock Vina: Improving the speed and accuracy of docking with a new scoring function, efficient optimization, and multithreading. J. Comput. Chem..

[26] Seeliger D., de Groot B.L. (2010). Ligand docking and binding site analysis with PyMOL and Autodock/Vina. J. Comput. Aided Mol. Des..

[27] Wang Y., Ryu B.H., Yoo W., Lee C.W., Kim K.K., Lee J.H., Kim T.D. (2018). Identification, characterization, immobilization, and mutational analysis of a novel acetylesterase with industrial potential (*La*AcE) from *Lactobacillus acidophilus*. Biochim. Biophys. Acta Gen. Subj..

[28] Lee C.W., Kwon S., Park S.H., Kim B.Y., Yoo W., Ryu B.H., Kim H.W., Shin S.C., Kim S., Park H. (2017). Crystal structure and functional characterization of an esterase (*Ea*EST) from *Exiguobacterium antarcticum*. PLoS ONE.

[29] Oh C., Ryu B.H., Yoo W., Nguyen D.D., Kim T., Ha S.C., Kim T.D., Kim K.K. (2017). Identification and Crystallization of Penicillin-Binding Protein/β-Lactamase Homolog (Rp46) from *Ruegeria Pomeroyi*. Crystals.

[30] Zottig X., Meddeb-Mouelhi F., Beauregard M. (2016). Development of a high-throughput liquid state assay for lipase activity using natural substrates and rhodamine B. Anal. Biochem..

[31] Cui J., Cui L., Jia S., Su Z., Zhang S. (2016). Hybrid cross-linked lipase aggregates with magnetic nanoparticles: A robust and recyclable biocatalysis for the epoxidation of oleic acid. J. Agric. Food Chem..

[32] Yoo W., Le L.T.H.L., Lee J.H., Kim K.K., Kim T.D. (2019). A novel enantioselective SGNH family esterase (NmSGNH1) from *Neisseria meningitides*: Characterization, mutational analysis, and ester synthesis. Biochim. Biophys. Acta Mol. Cell. Biol. Lipids..

[33] Yoon S., Kim S., Ryu Y., Kim T.D. (2007). Identification and characterization of a novel (S)-ketoprofen-specific esterase. Int. J. Biol. Macromol..

[34] Wagner U.G., Petersen E.I., Schwab H., Kratky C. (2002). EstB from *Burkholderia gladioli*: A novel esterase with a beta-lactamase fold reveals steric factors to discriminate between esterolytic and beta-lactam cleaving activity. Protein Sci..

[35] Cha S.S., An Y.J., Jeong C.S., Kim M.K., Jeon J.H., Lee C.M., Lee H.S., Kang S.G., Lee J.H. (2013). Structural basis for the β-lactamase activity of EstU1, a family VIII carboxylesterase. Proteins.

[36] Wouters J., Fonze E., Vermeire M., Frere J.M., Charlier P. (2003). Crystal structure of *Enterobacter cloacae* 908R class C beta-lactamase bound to iodoacetamido phenyl boronic acid, a transition-state analogue. Cell. Mol. Life Sci..

[37] Oguri T., Furuyama T., Okuno T., Ishii Y., Tateda K., Bonomo R.A., Shimizu-Ibuka A. (2014). Crystal structure of Mox-1, a unique plasmid-mediated class C β-lactamase with hydrolytic activity towards moxalactam. Antimicrob. Agents. Chemother..

[38] Schutte M., Fetzner S. (2007). EstA from *Arthrobacter nitroguajacolicus* Rü61a, a thermo- and solvent-tolerant carboxylesterase related to class C beta-lactamases. Curr. Microbiol..

[39] Kim S., Joo S., Yoon S., Kim S., Moon J., Ryu Y., Kim K.K., Kim T.D. (2009). Purification, crystallization and preliminary crystallographic analysis of Est-Y29: A novel oligomeric beta-lactamase. Acta Crystallogr. Sect. F Struct. Biol. Cryst. Commun..

[40] Rashamuse K.J., Burton S.G., Stafford W.H., Cowan D.A. (2007). Molecular characterization of a novel family VIII esterase from *Burkholderia multivorans* UWC10. J. Mol. Microbiol. Biotechnol..

[41] Oh C., Ryu B.H., Yoo W., Nguyen D.D., Kim T., Ha S.-C., Kim T.D., Kim K.K. (2018). Identification and Crystallographic Analysis of a New Carbohydrate Acetylesterase (SmAcE1) from *Sinorhizobium meliloti*. Crystals.

[42] Jeon J.H., Kim S.J., Lee H.S., Cha S.S., Lee J.H., Yoon S.H., Koo B.S., Lee C.M., Choi S.H., Lee S.H. (2011). Novel metagenome-derived carboxylesterase that hydrolyzes β-lactam antibiotics. Appl. Environ. Microbiol..

[43] Yu E.Y., Kwon M.A., Lee M., Oh J.Y., Choi J.E., Lee J.Y., Song B.K., Hahm D.H., Song J.K. (2011). Isolation and characterization of cold-active family VIII esterases from an arctic soil metagenome. Appl. Microbiol. Biotechnol..

[44] Sirisha V.L., Jain A., Jain A. (2016). Enzyme immobilization: An overview on methods, support material, and applications of immobilized enzymes. Adv. Food. Nutr. Res..

[45] Di Cosimo R., McAuliffe J., Poulose A.J., Bohlmann G. (2013). Industrial use of immobilized enzymes. Chem. Soc. Rev..

[46] Sheldon R.A. (2011). Characteristic features and biotechnological applications of cross-linked enzyme aggregates (CLEAs). Appl. Microbiol. Biotechnol..

[47] Altinkaynak C., Tavlasoglu S., Özdemir N., Ocsoy I. (2016). A new generation approach in enzyme immobilization: Organic-inorganic hybrid nanoflowers with enhanced catalytic activity and stability. Enzyme Microb. Technol..

[48] Lee S.W., Cheon S.A., Kim M.I., Park T.J. (2015). Organic-inorganic hybrid nanoflowers: Types, characteristics, and future prospects. J. Nanobiotechnology.

[49] Wagner U.G., DiMaio F., Kolkenbrock S., Fetzner S. (2014). Crystal structure analysis of EstA from *Arthrobacter* sp. Rue61a—An insight into catalytic promiscuity. FEBS Lett..

[50] Littlechild J.A. (2015). Enzymes from extreme environments and their industrial applications. Front. Bioeng. Biotechnol..

[51] Mitusińska K., Magdziarz T., Bzówka M., Stańczak A., Góra A. (2018). Exploring *Solanum tuberosum* Epoxide Hydrolase Internal Architecture by Water Molecules Tracking. Biomolecules.

